# Supplementary material for: Seminoma and Embryonal Carcinoma Footprints Identified by Analysis of Integrated Genome-Wide Epigenetic and Expression Profiles of Germ Cell Cancer Cell Lines
Source: PLoS One. 2014 Jun 2;9(6):e98330. doi: 10.1371/journal.pone.0098330 (PMC4041891; doi:10.1371/journal.pone.0098330)
Supplement: File S1 — ZIP file containing DMRforPairs output for significant regions. Please start from the html files. (ZIP) [file pone.0098330.s008.zip › figures/31437.pdf]

RegionID: 31437, chr4:188916496−188916943−M_values

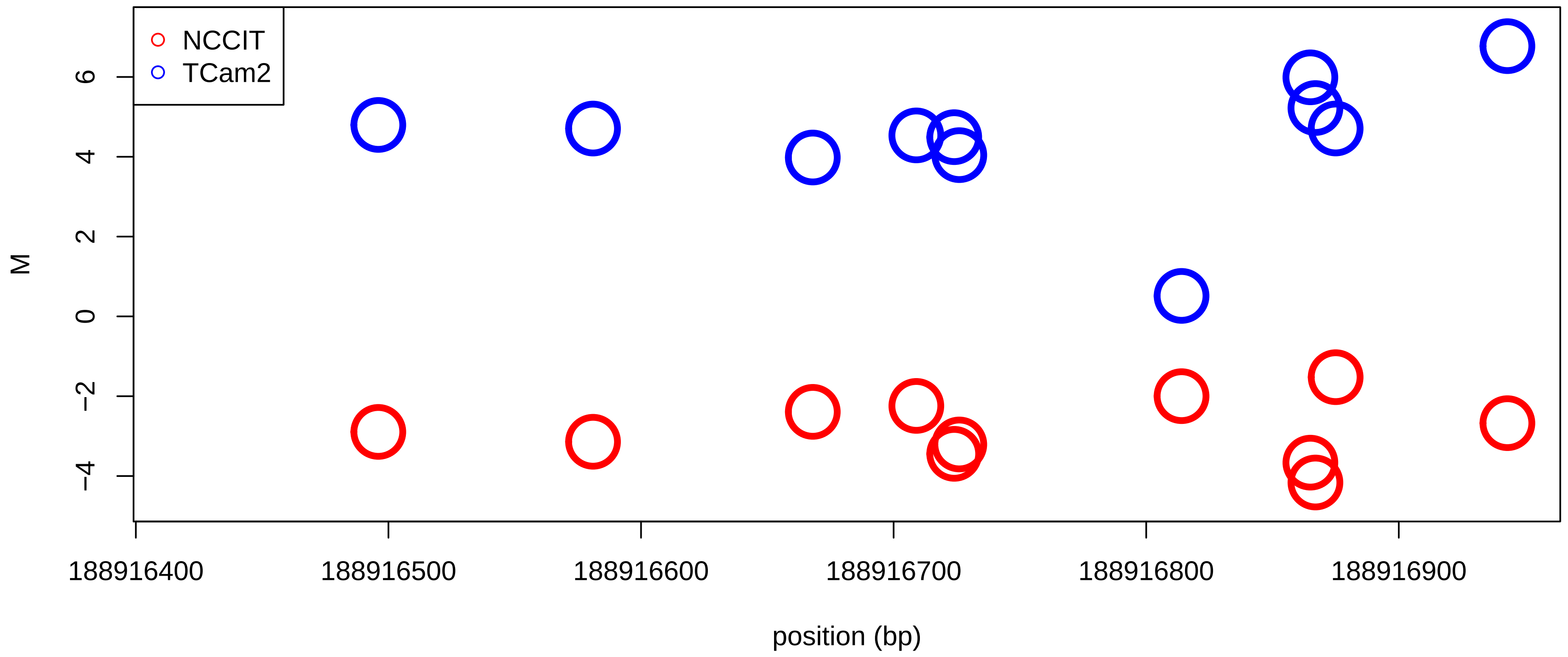


RegionID: 31437, chr4:188916496−188916943−Beta_values

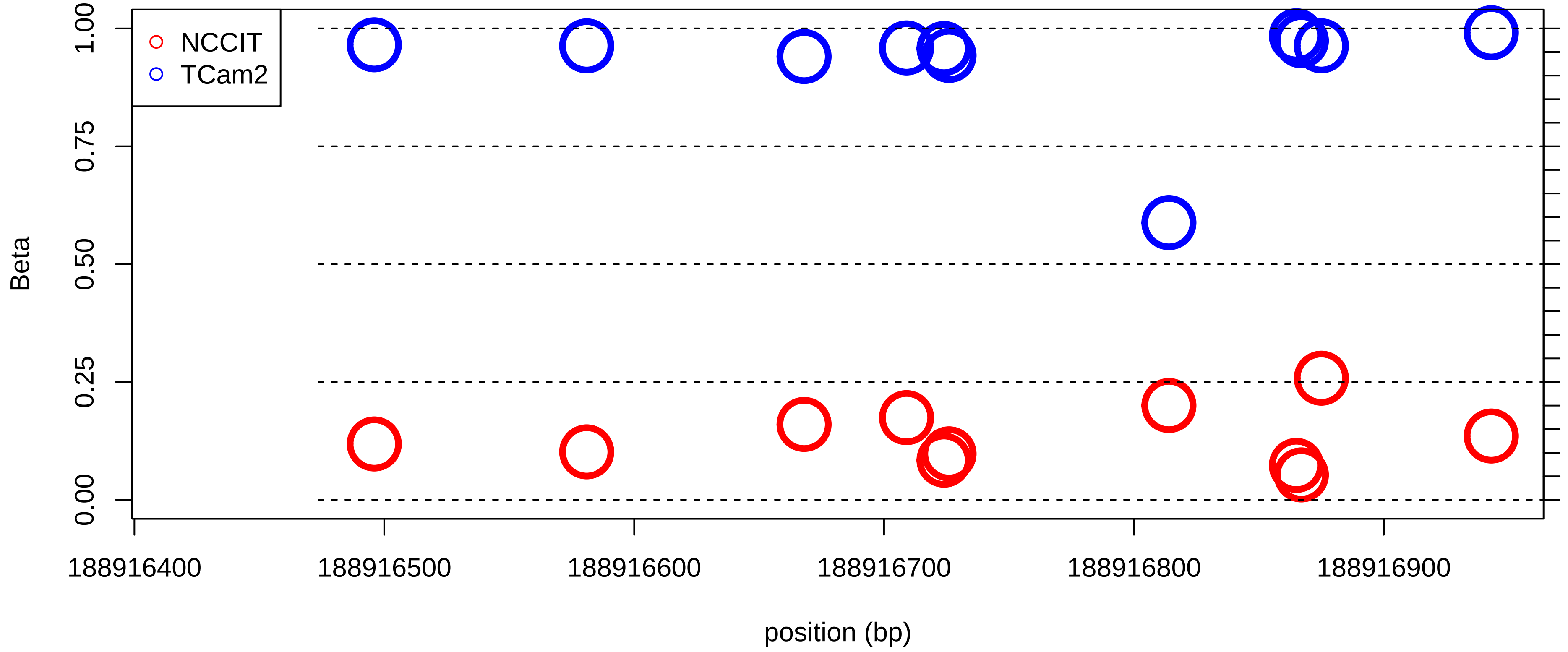

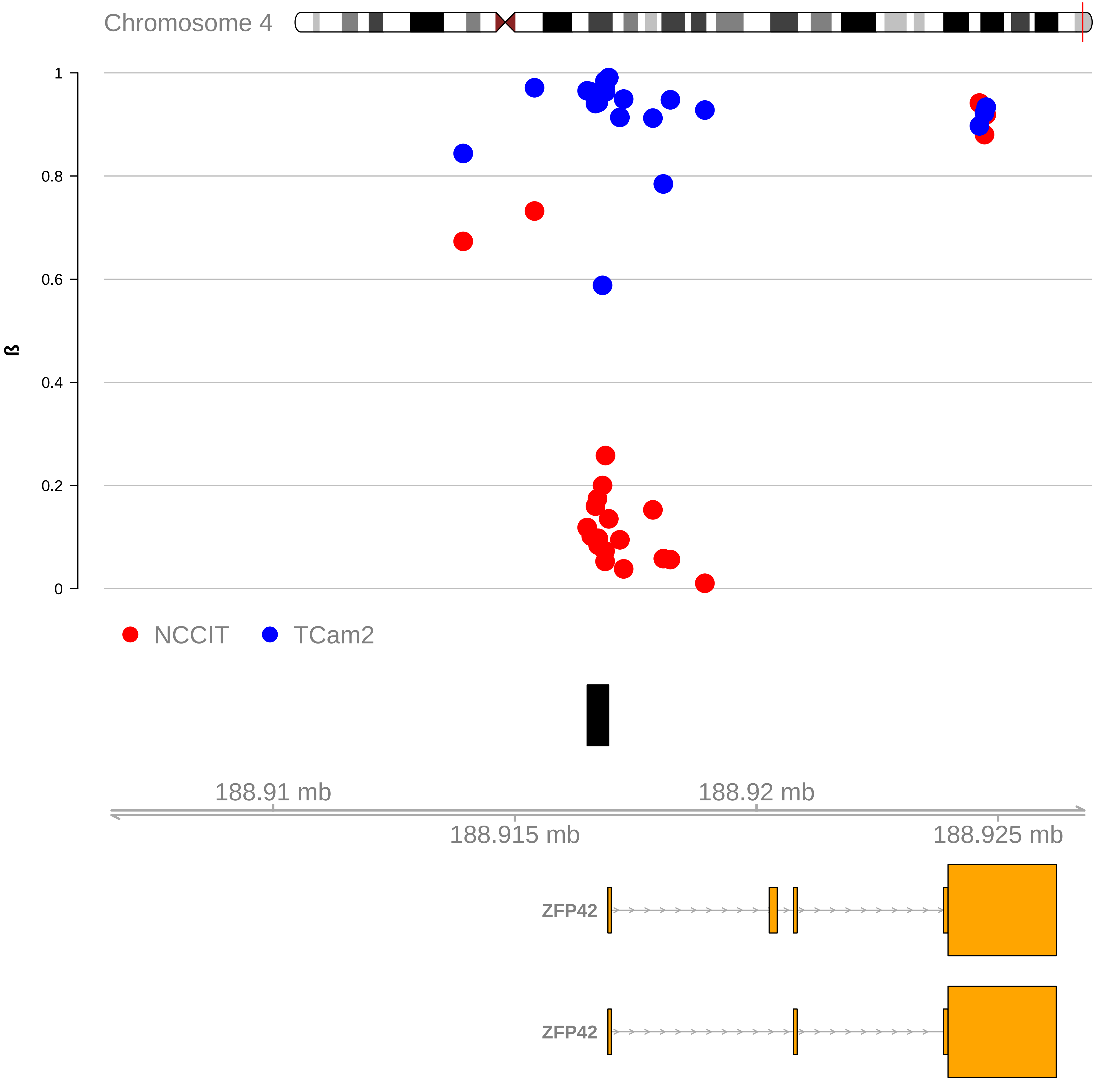
Chromosome 4
1
0.8
0.6
0.4
0.2
0
ß
NCCIT
TCam2
188.91 mb
188.92 mb
188.915 mb
188.925 mb
ZFP42
ZFP42
